# Coping strategies following the diagnosis of a fetal anomaly: A scoping review

**DOI:** 10.3389/fpubh.2023.1055562

**Published:** 2023-04-06

**Authors:** Tingting Zhang, Wei-Ti Chen, Qingnan He, Ying Li, Huiting Peng, Jiaying Xie, Hengfen Hu, Chunxiang Qin

**Affiliations:** ^1^Department of Health Management, The Third Xiangya Hospital and Xiangya School of Nursing, Central South University, Changsha, China; ^2^Xiangya School of Nursing, Central South University, Changsha, Hunan, China; ^3^School of Nursing, University of California, Los Angeles, Los Angeles, CA, United States; ^4^Pediatric Department of the Third Xiangya Hospital, Central South University, Changsha, Hunan, China; ^5^Hunan Polytechnic of Environment and Biology, Hengyang, Hunan, China

**Keywords:** fetal anomaly, mental distress, scoping review, coping strategies, women's health

## Abstract

**Introduction:**

Many women experience severe emotional distress (such as grief, depression, and anxiety) following a diagnosis of fetal anomaly. The ability to cope with stressful events and regulate emotions across diverse situations may play a primary role in psychological wellbeing. This study aims to present coping strategies after disclosing a fetal anomaly to pregnant women.

**Methods:**

This is a scoping review based on the Preferred Reporting Items for Systematic Reviews and Meta-Analyses (PRISMA) extension for scoping reviews (PRISMA-ScR). Electronic databases, including Web of Science (WOS, BCI, KJD, MEDLINE, RSCI, SCIELO), CINAHL, and EBSCO PsycARTICLES, were used to search for primary studies from the inception of each database to 2021. The keywords were determined by existing literature and included: “fetal anomaly,” “fetal abnormality,” “fetal anomaly,” “fetal abnormality” AND “cope,” “coping,” “deal,” “manage,” “adapt^*^,” “emotion^*^ regulate^*^,” with the use of Boolean operators AND/OR. A total of 16 articles were reviewed, followed by advancing scoping review methodology of Arksey and O'Malley's framework.

**Results:**

In this review, we identified 52 coping strategies using five questionnaires in seven quantitative studies and one mixed-method study. The relationship between coping strategies and mental distress was explored. However, the results were inconsistent and incomparable. We synthesized four coping categories from qualitative studies and presented them in an intersection.

**Conclusion:**

This scoping review identified the coping strategies of women with a diagnosis of a fetal anomaly during pregnancy. The relationship between coping strategies and mental distress was uncertain and needs more exploration. We considered an appropriate measurement should be necessary for the research of coping in women diagnosed with fetal anomaly pregnancy.

## Introduction

Screening for fetal anomalies is part of prenatal care, which is recommended for all pregnant women worldwide (1). The diagnosis of fetal anomalies is occurring more frequently because of the increasing use of sophisticated obstetric technologies. The overall prevalence of fetal anomalies was 2.09% in a Cochrane review (2) and ~3.04% in China (3). This diagnosis is a major source of stress for women and their families, resulting in mental distress, such as grief, depression, and anxiety (4–11). While dealing with the grieving experience due to the abnormality, women concurrently manage other stressful events, such as decision-making on continuing or terminating the pregnancy (12–14). Research has indicated that their symptoms of grief were more serious than women with normal pregnancies, even amid the COVID-19 pandemic (15), and persisted for several years after being diagnosed with a fetal anomaly pregnancy (16, 17).

Coping is defined as constantly changing cognitive and behavioral efforts to manage (reduce, minimize, master, or tolerate) specific external and/or internal demands that are appraised as taxing or exceeding the resources of the person (18, 19). Generally, coping with stressful events plays a primary role in controlling the situation for reducing mental distress (20, 21). Coping plays an important role in the relationship between stress and mental health (22). Different coping strategies bring different mental health outcomes, such as self-distraction appearing to be associated with lower psychological distress and avoidant coping and denial associated with increased psychological distress (23). Lazarus and Folkman's stress and coping theory identified two processes, cognitive appraisal and coping, as critical mediators of stressful person–environment transactions (18, 19).

In studies of coping, researchers reported many kinds of coping strategies, and the instruments to evaluate coping strategies were inconsistent (24–26). On their path to analyzing categories and systems of coping, Skinner et al. identified approximately 400 coping techniques and approximately 100 schemes to classify coping (24). Among these categories, engagement vs. disengagement and primary control vs. secondary control were used in a multidimensional model to be higher-order coping categories (24, 27). Engagement responses are directed toward the stressor or one's reactions to the stressor and include approach responses; disengagement responses are oriented away from a stressor or one's reactions and include avoidance responses. Primary control refers to coping attempts that are directed toward influencing objective events or conditions; secondary control coping involves efforts to fit with or adapt to the environment (27, 28). Some other researchers also tried to present the structure of coping strategies more clearly using hierarchical models; for example, Trawalter et al. adapted a framework from cognitive appraisals to the coping behavior of interracial contact (29). Mara et al. used the intersection of two elements (personal empowerment vs. collective empowerment: disclosure vs. concealment), which resulted in four coping strategies (reactive, proactive, internalize, and externalize) (30). Furthermore, Ewert et al. showed the coping structure with a three-level pyramid; the first level was adaptive and maladaptive coping, the second level was problem-focused and emotion-focused coping, and the third level was individual coping strategies (31). However, articles presented a lack of gold standards for evaluating coping strategies (25, 26).

Situational factors, which refer to the objective features of the event, and the person's appraisal (primary and secondary appraisal) of the situation play as determinants of coping (18, 19, 32). The effectiveness of any coping strategy and its impact on wellbeing may vary from situation to situation (33, 34). Therefore, women will use different coping responses from other stressful events when diagnosed with fetal anomaly pregnancy. Their coping is a critical mediator between the event and their mental distress. Various methods were used to explore women's coping strategies in the situation of being diagnosed with fetal anomaly pregnancy, but relative reviews were underreported. A scoping review will be helpful for summarizing research findings, and identifying research gaps in the existing literature (35, 36), and supporting professional practices and new research on the subject.

## Methods

### Objectives

In this study, we are aiming to review the coping strategies when a woman is diagnosed with fetal anomaly pregnancy. This review formed two study questions based on the population, concept, and context (PCC) elements (37): (1) “What types of coping strategies have been reported when women are diagnosed with fetal anomaly pregnancy? and (2) what is the influence of coping strategies upon mental distress, including grief, depression, or anxiety, in this situation?” [SIC] We present the following article in accordance with the PRISMA-ScR reporting checklist.

### Design

This is a scoping review including evidence from qualitative, quantitative, and mixed-method studies. Five steps of the advancing methodology of Arksey and O'Malley's framework (35, 36, 38) are used as follows: (1) identifying the research question, (2) identifying relevant studies, (3) study selection and assessment, (4) charting the data, and (5) collating, summarizing, and reporting results.

### Search methods

#### Inclusion/exclusion criteria

Studies were included based on the following inclusion criteria: (1) Studies that reported on the coping strategies of women with a diagnosis of a fetal anomaly during pregnancy; (2) data were collected from the women with a diagnosis of a fetal anomaly during pregnancy, their partners, or their healthcare providers; and (3) primary studies published in English or Chinese from the inception of each database to 2021. Exclusion criteria included the following: (1) review articles and (2) other languages other than English and Chinese.

#### Search strategies and study selection

**Figure 1:**
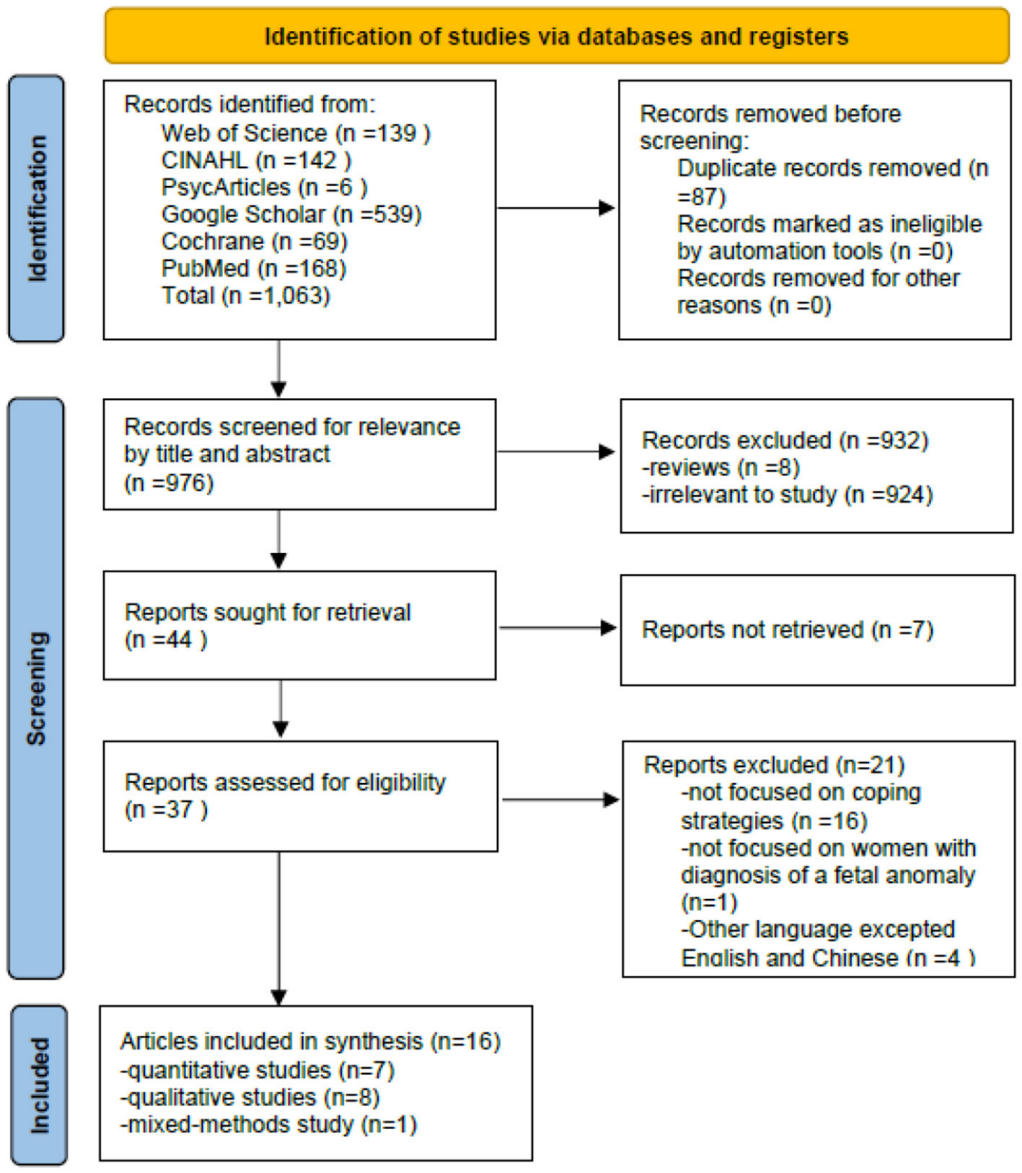
Flow diagram of the article.

The following databases were used to search for primary studies: Web of Science (WOS, BCI, KJD, MEDLINE, RSCI, SCIELO), CINAHL and EBSCO, PsycARTICLES, PubMed, Google Scholar, and Cochrane. The initial literature search was performed in September 2020, and the search was updated in December 2020 and February 2023. The keywords were determined from existing literature and included: “fetal anomaly,” “fetal abnormality,” “fetal anomaly,” “fetal abnormality” AND “cope,” “coping,” “deal,” “manage,” “adapt^*^,” “emotion^*^ regulate^*^,” with the use of Boolean operators AND/OR. See Table 1 for an example of search terms from one database and the yielded numbers. The screening process included four steps and is shown in Figure 1. First, all titles and abstracts retrieved were downloaded to the Endnote X9 library, and duplicate articles were removed. Second, articles were excluded based on titles and abstracts. Third, the full text of the article was reviewed to determine eligibility based on the inclusion and exclusion criteria. Finally, the reference lists of the included articles and related reviews were screened for potentially relevant articles. The searching and screening processes were conducted by two independent researchers, and disagreements were resolved by a third researcher.

**Table 1:** Example of search strategy Web of science.

**Main concept**	**Search formula**	**Results**
1. Fetal anomaly	(((TS=(“Fetal Malformation^*^”)) OR TS=(“Fetal Anomal^*^”)) OR TS=(“Malformation, Fetal”)) OR TS=(“Anomaly, Fetal”)	6,451
2. Coping	(((((((((((((TS=(“Psychologic^*^ Adaptation”)) OR TS=(“Adaptation, Psychologic”)) OR TS=(Adjustment)) OR TS=(“mind^*^”)) OR TS=(“Coping Behavior^*^”)) OR TS=(“Behavior^*^, Coping”)) OR TS=(“Coping Skill^*^”)) OR TS=(“Skill^*^, Coping”)) OR TS=(“Coping Strateg^*^”)) OR TS=(“Strateg^*^, Coping”)) OR TS=(“Behavior^*^, Adaptive”)) OR TS=(“Adaptive Behavior^*^”)) OR TS=(“Emotion^*^ Regulation^*^”)) OR TS=(“Regulation^*^, Emotion^*^”)	786,637
#1 AND #2		132

### Quality appraisal

**Figure 2:**
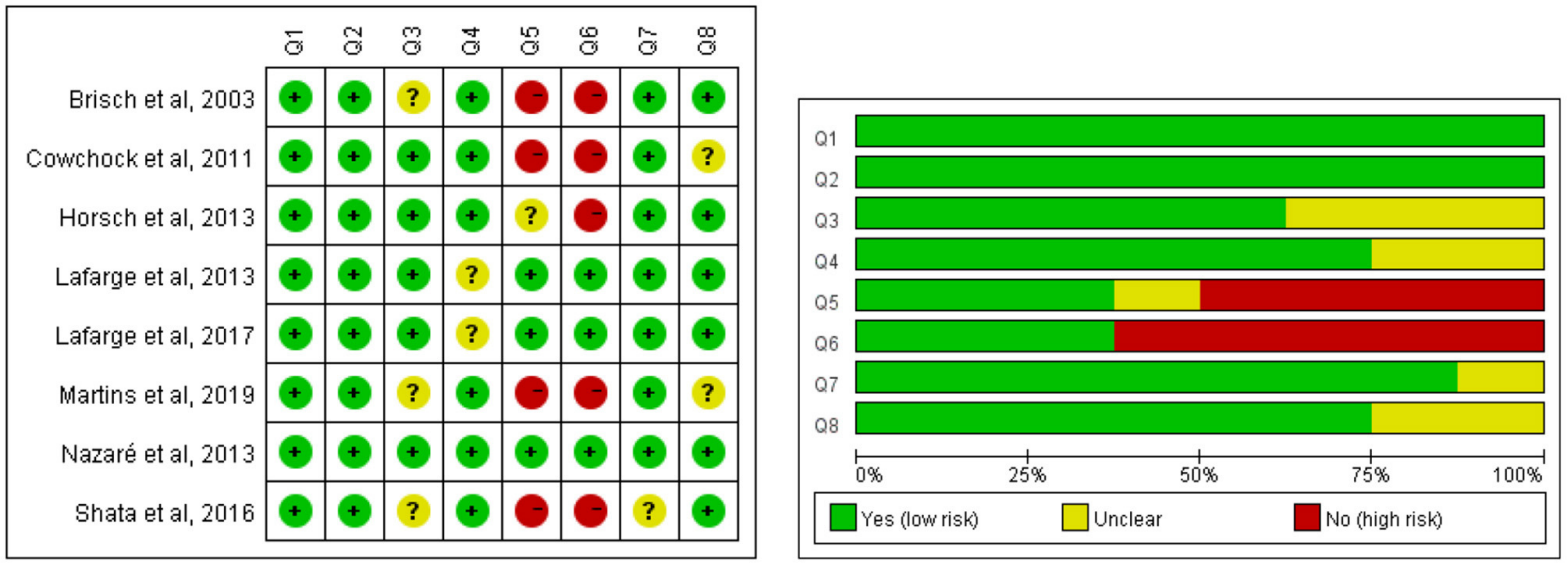
Methodological quality of quantitative studies. Q1: Were the criteria for inclusion in the sample clearly defined? Q2: Were the study subjects and the setting described in detail? Q3: Was the exposure measured in a valid and reliable way? Q4: Were objective, standard criteria used for measurement of the condition? Q5: Were confounding factors identified? Q6: Were strategies to deal with confounding factors stated? Q7: Were the outcomes measured in a valid and reliable way? Q8: Was appropriate statistical analysis used?

**Figure 3:**
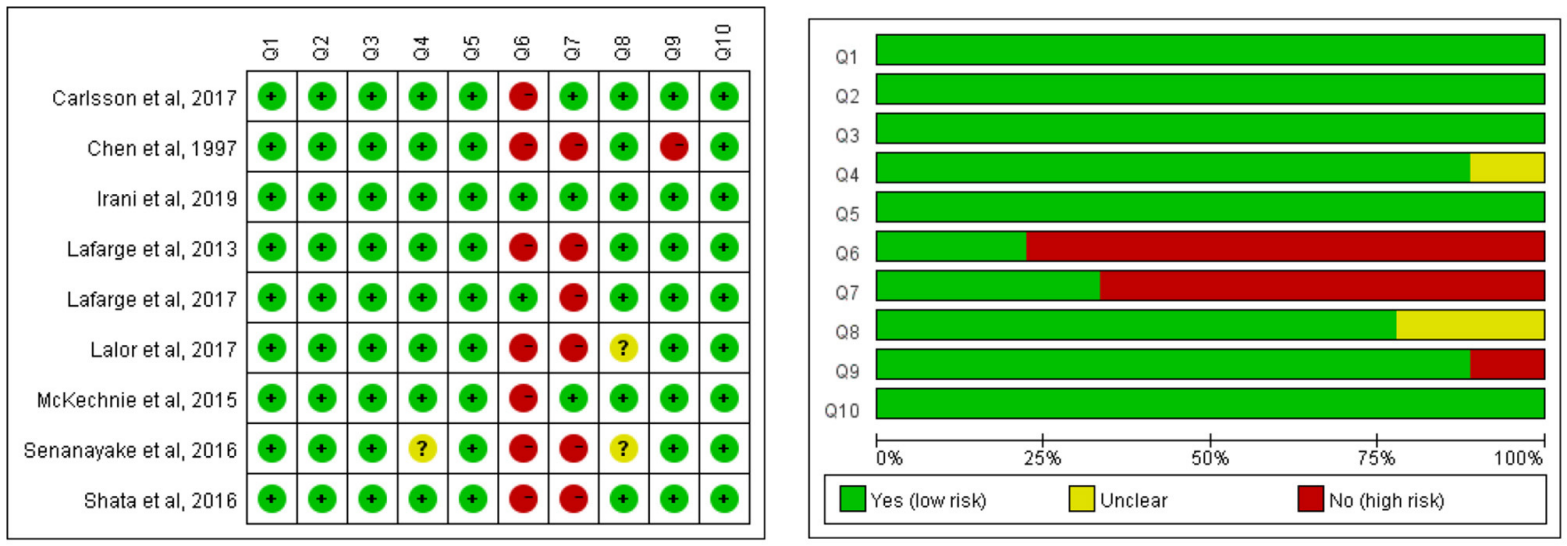
Methodological quality of qualitative studies. Q1: Was there congruity between the stated philosophical perspective and the research methodology? Q2: Was there congruity between the research methodology and the research question or objectives? Q3: Was there congruity between the research methodology and the methods used to collect data? Q4: Was there congruity between the research methodology and the representation and analysis of data? Q5: Was there congruity between the research methodology and the interpretation of results? Q6: Was there a statement locating the researcher culturally or theoretically? Q7: Was the influence of the researcher on the research, and vice-versa, addressed? Q8: Were participants, and their voices, adequately represented? Q9: Was the research ethical according to current criteria or, for recent studies, and is there evidence of ethical approval by an appropriate body? Q10: Did the conclusions drawn in the research report flow from the analysis, or interpretation, of the data?

The quality of the included articles was assessed by two independent researchers using the Joanna Briggs Institute Critical Appraisal checklist for analytical cross-sectional studies (Figure 2) and qualitative research (Figure 3). The mix-method study was separately appraised by both analytical cross-sectional studies and qualitative research checklists. Each entry was answered with yes, no, or unclear. As the review sought to explore the totality of evidence relating to coping strategies after being diagnosed with fetal anomaly pregnancy, no disqualifications were made on the quality evaluation process. The quality evaluating process only assisted in building a picture of the current study characteristic of the field.

### Data extraction

Data from each study were extracted using an Excel database (Table 2). Next, two reviewers read the articles carefully and then extracted the data. The extracted content included the title, author, year, country, aim, methodology/design, sample strategy and sample size, data collection method, analytical approach, and summary of main findings.

**Table 2:** Individual characteristics of the included studies (*n* = 16) on coping strategies of the women diagnosed with a fetal anomaly.

**Title, author, publication year, and country**	**Study aim**	**Methodology/ design**	**Recruitment and sample size**	**Data collection method**	**Analytical approach**	**Summary of main findings and limitations**
Influence of Religiosity on Situational Coping Scores in Women with Malformed Fetuses. Martins et al. (39), Brazil	To evaluate the influence of religiosity on the situational coping mechanisms of pregnant women with fetal abnormalities.	A descriptive cross-sectional quantitative study	Women attending the High-Risk Pregnant Division of FMRP-USP with a diagnosis of fetal anomaly (*n* = 28)	Questionnaire (CBPS)	Spearman's rank correlation coefficient test and Fisher's exact test	Main findings: religious practices were associated with improved coping in women diagnosed with fetal abnormalities and should be encouraged in care settings. Limitations: not reported
Coping Strategies of Pregnant Women with Detected Fetal Anomalies in Irani: A Qualitative Study. Irani et al. (40), Iran	To explore coping strategies of pregnant women after the detection of fetal anomalies.	A descriptive qualitative study	Persian speaking pregnant women with prenatal diagnosis of fetal anomaly (*n* = 25)	Individual semi-structured interviews	content analysis	Main findings: the findings showed that pregnant women with detected fetal anomalies reported a variety of coping strategies: seeking information, religiousness and spirituality, cognitive avoidance, and seeking social support. Limitations: participants were heterogeneous; data were not longitudinal
Post-traumatic growth following pregnancy termination for fetal abnormality: the predictive role of coping strategies and perinatal grief. Lafarge et al. (4), UK	To measure PTG following TFA, examine the relationship between PTG, perinatal grief and coping, determine the predictors of PTG.	Online cross-sectional survey	Women over 18 years old who had undergone TFA of a UK-based support organization through online message posted (*n* = 161)	Questionnaire (PTGI, COPE and PGS)	*Post-hoc* test; *t*-tests; one-way analysis of variance test; correlation; multiple regression analyses	Main findings: “Positive reframing” was a significant predictor of PTG. Despite using mainly “adaptive” coping strategies, women's grief levels were high. Limitations: data were not longitudinal; participants limited; a social desirability bias
Pregnancy termination for fetal abnormality: are health professionals' perceptions of women's coping congruent with women's accounts? Lafarge et al. (4), UK	To examine health professionals' perceptions of women' scoping with TFA and compare them to women's accounts to assess their congruence.	An interpretative qualitative research	Any health professionals involved in the pregnancy management of women including consultants, midwives, and sonographers (*n* = 14)	Face-to-face semi-structured interviews	Thematic analysis	Main findings: The findings indicate that health professionals' perceptions of women's coping covered six areas that were also present in women's accounts: “support,” “acceptance,” “problem solving,” “avoidance,” “another pregnancy” and “meaning attribution” Limitations: participants recruited mostly in two hospitals with a large fetal medicine center in England; sample was self-selected; social desirability bias
The emotional process from diagnosis to birth following a prenatal diagnosis of fetal anomaly: A qualitative study of messages in online discussion boards. Carlsson et al. (41), Sweden	To explore written statements found in online discussion boards about their emotional process from diagnosis to birth.	A qualitative study of messages based on grounded theory	posters and messages from (1) be in Swedish, (2) be public and freely accessible *via* the Web, (3) include sections about reproductive issues, and (4) include the option to write anonymously. (18 posters and 852 messages)	Search in Google	Constant comparative method by NVivo	Main findings: three phases were identified in the process of moving from the diagnosis to the birth: shock, existential crisis, and life remodeling. To cope with their situation, the posters distanced themselves from the diagnoses, vented their feelings, sought control, and obtained practical support from friends and relatives. Limitations: demographics of internet users may differ from those of the entire population; Internet users may have different experiences or characteristics from those do not write online; written messages may leave the experiences related open to a risk of misinterpretation.
Coping strategies of pregnant women following unexpected ultrasound results, Alexandria, Egypt. Shata et al. (42), Egypt	To explore how woman cope with stress following unexpected fetal diagnoses during routine antenatal ultrasound scan	A mixed-method (quantitative and qualitative study)	Targeted pregnant women at 20–32 weeks of gestation who would get fetuses with congenital anomalies for quantitative (*n* = 93), purposive sampling for qualitative (*n* = 30)	Questionnaire (revised from COPE) and semi-structured interview.	One-sample Kolmogorov–Smirnov test; Friedman test; Wilcoxon's signed-rank test; w2 and Fisher's exact tests; thematic analysis	Main findings: Women concurrently used more than one coping strategy. The effectiveness of active strategies was higher than that of the avoidant ones. The educational level was the only factor associated with perceived high effectiveness of active and/or avoidant strategies. Limitations: data were not longitudinal; data belong only to women attending the two settings; future large-scale studies are necessary
Preparing Heart and Mind for Becoming a Parent Following a Diagnosis of Fetal Anomaly. McKechnie et al. (43), USA	To address the gap in the theoretical and empirical literature by examining how parenting develops after a major fetal an female, over 18 years old and had undergone TFA omaly diagnosis	A ground theory study	Convenience sampling to recruit expectant parents who received a diagnosis of a major fetal anomaly and made a decision to continue pregnancy from two regional tertiary care centers (25 pregnant women and 12 men)	An unstructured, interactive interview	Grounded dimensional analysis	Main findings: this study described a theoretical model with a core process of preparing heart and mind for becoming a parent following a diagnosis of fetal anomaly. Expectant parents reported varying turning points and strategies associated with three distinct trajectories of relating to the fetus or “baby” yet to be born. Limitations: data were not longitudinal; disproportionate number of expectant mothers to expectant fathers; response bias
Adaptive and maladaptive grief responses following TOPFA: actor and partner effects of coping strategies. Nazare et al. (44), Portugal	To (1) compare women and men in the first 6 months following TOPFA regarding absolute and relative coping; and (2) assess the influence of relative coping on each partner's adaptive and maladaptive grief responses.	A longitudinal investigation	Convenience sample of couples having experienced TOPFA 1–6 months earlier, being 18 years or older, and having a level of literacy (*n* = 41)	Questionnaire (COPE and PGS)	Repeated-measures MANOVAs; Pearson correlations; multiple linear regressions	Main findings: Women used Religion more frequently than men. Women's absolute and relative scores on Emotional Support, Instrumental Support and Venting were higher than men's. Four coping strategies were proved unhelpful: self-blame, denial, men's relative use of instrumental support and partner effects of active coping. Two strategies emerged as helpful: women's relative use of acceptance and humor. Limitations: small sample; data were not longitudinal; four Brief COPE subscales were excluded
Perinatal grief following a termination of pregnancy for fetal abnormality: the impact of coping strategies. Lafarge et al. (6), UK	To examine the relationships between women's coping strategies and perinatal grief.	A cross-sectional survey	Female, over 18 years old and had undergone TFA recruited through network and forum (*n* = 166)	Online survey, questionnaire (COPE and PGS)	Bonferroni *post-hoc* test (equal variances); *t*-tests; one-way analysis of variance test; Multiple hierarchical regressions	Main findings: women having had a TFA relied more on adaptive than maladaptive coping strategies. women who reported using strategies such as acceptance and positive reframing fared better psychologically than those who used more maladaptive strategies such as self-blame or behavioral disengagement. The high levels of grief in the presence of adaptive coping strategies also lead us to question the validity of classifying coping strategies into distinct categories either as adaptive or maladaptive Limitations: data were not longitudinal; participant profile was strikingly predominantly White, well-educated;
Women's Experiences of Coping With Pregnancy Termination for Fetal Abnormality. Lafarge et al. (6), UK	To examine the coping strategies women use both during and after a termination for fetal abnormality procedure.	A cross-sectional retrospective interpretative qualitative interview	Members of a support group based in the United Kingdom that provided support to parents with fetal anomaly through the group's email network and forum (*n* = 27)	Collect data online through qualitative interview schedule consisted of open-ended questions	Interpretative phenomenological analysis	Main findings: Coping comprised four structures, consistent across time points: support, acceptance, avoidance, and meaning attribution. Women mostly used adaptive coping strategies but reported inadequacies in aftercare, which challenged their resources. Limitations: sample was limited to women who were active members of a support group; social desirability bias
Maternal coping, appraisals and adjustment following diagnosis of fetal anomaly. Horsch et al. (7), UK	identify predictors of coping and later adjustment and to understand the role of appraisals in women who continue with their pregnancy following a diagnosis of fetal anomaly.	A retrospective investigation	Women over the age of 16 years; received a diagnosis of fetal anomaly in a prenatal diagnosis unit of an NHS university hospital (*n* = 40)	Questionnaire (HADS, ABS, PDS, PSI, SSQ6, ALES, Coping Options, COPE)	Mann–Whitney U or Wilcoxon signed-rank tests; Fisher's exact test; chi-square tests; two hierarchical stepwise regressions	Main findings: Participants used both emotion-focused and problem-focused strategies, and both were associated with improved adjustment. Limitations: sample size was small; participants were recruited online; questionnaires were completed retrospectively
Religiosity is an Important Part of Coping with Grief in Pregnancy After a Traumatic Second Trimester Loss. Cowchock et al. (45), USA	To evaluate the frequency of such distress, including depression, anxiety, and PTS symptoms, and investigate the role of potential predictors such as religiosity and religious attendance.	A pilot survey	Spoke English, and had terminated a prior pregnancy after the first trimester at Duke University Medical Center (*n* = 15)	Questionnaire (Hoge Scale, PGS, IES, DDI)	Statistics was unclear	Main findings: high levels of grief and PTS symptoms are significant problems for pregnant women who have suffered late loss of a wanted pregnancy. Religiosity may play an important part in maternal coping during these stressful pregnancies. Limitations: not reported
A grounded theory study of information preference and coping styles following antenatal diagnosis of fetal abnormality. Lalor et al. (46), Ireland	To offer a theoretical perspective on the interacting effects of information preference in coping with an adverse prenatal diagnosis.	A longitudinal ground theory study	Women with low risk of pregnancy complications receiving a diagnosis of fetal anomaly (*n* = 42)	Interview with more focused as theoretical sampling	constant comparative method	Main findings: Two main categories were identified: ‘Getting my head around it' and ‘I'll cross that bridge when I come to it'. These two different information-seeking preferences are described as monitoring and blunting. Limitations: the context is very specific;
Psychological reactions and coping strategies of Sri Lankan women carrying fetuses with lethal congenital malformations. Senanayake et al. (47), Sri Lanka	To describe the psychological reactions and coping strategies of women who were knowingly carrying fetuses with lethal congenital abnormalities.	A descriptive qualitative research	women whose fetuses were diagnosed as lethal congenital abnormality and who were receiving care at the Professorial Obstetric Unit of the University of Colombo (*n* = 10)	Semi-structured interview	unclear	Main findings: the diagnosis of LCM causes severe distress and psychological reactions. The women need minimum contact with other women, taking into account the futility of the pregnancy, engaging in religious rites, even though with unreal expectations. Limitations: not reported
Coping styles of pregnant women after prenatal ultrasound screening for fetal malformation. Brisch et al. (8), Germany	To investigate the coping process of women with a risk for fetal malformation detected by ultrasound scanning.	Prospective longitudinal study	Women of the high-risk group detected indices of a fetal malformation and advised the women to go to the University Center for a further examination. (*n* = 664) 497 in high-risk group and 167 in control group	Questionnaire (German version of a questionnaire on coping process, STAI)	Chi-square test; *T*-test; Mann–Whitney U-test, ANOVA; Kruskal–Wallis H-test; General Linear Model; MANOVA	Main findings: Women with risk pregnancies used coping strategies similar to those women in the control group. Different spectrums of coping strategies corresponded significantly to increasing or decreasing anxiety. Limitations: not reported
Life experience and coping behavior of a woman receiving termination of pregnancy due to fetal abnormality in the second trimester of pregnancy. Chen et al. (48), China	To explore the life experience and coping behavior of a woman after receiving the diagnosis of fetal anomaly.	A descriptive qualitative research	One woman who terminated her pregnancy due to fetal anomaly	Field method	Content analysis	Main findings: three main areas of life experience, several coping behaviors including religious beliefs, accepting fate, rationalizing, avoiding and recommending maternal serum screening. Limitations: not reported

### Synthesis

The meta-ethnographic approach was used for qualitative synthesis in which the themes of individual studies are contrasted and compared systematically (49, 50). Qualitative synthesis identifies congruence and convergence in concepts and themes across individual studies, leading to new insights through secondary analysis and reinterpretation. It can be broken down into the following two stages; (a) reciprocal and refutational synthesis and (b) line of argument synthesis (50). A line of argument synthesis is achieved by constant comparison of the concepts and developing a ‘grounded theory that puts the similarities and differences between the studies into interpretative order' (50). Constant comparison with previous literature and theoretical frameworks is necessary for expanding and exceeding the theoretical framework (51).

## Results

### Overview of study characteristics

A flow diagram is used to present the results of the search process (Figure 1); a total of 1,063 studies were identified by searching six databases. Duplicate references were identified and removed (*N* = 87). After examining titles and abstracts, 932 full texts were further screened. Then, 28 studies were excluded for not being retrievable, not focused on our topic, or other languages, and 16 studies were identified as meeting our criteria and were included in this review. The characteristics of these studies are shown in Table 3. These 16 studies come from 11 countries. The participants are women, their partners, or health professionals. Six studies in all studies recruited participants who terminated and continued pregnancy, seven termination restrictions, and three restrictions continued. A place for data collection is a care setting, online, or a convenient place for participants. The content of the included studies examining the coping strategies of women diagnosed with fetal anomaly pregnancy is presented in Table 2. Qualitative studies (*n* = 8), quantitative studies (*n* = 7), and mixed-method studies (*n* = 1) are presented. The total number of participants in the studies was 1,215.

**Table 3:** Characteristics of included studies (*n* = 16).

**Characteristics**		**Characteristics**	
Country (Totals)	16	Participants (Totals)	16
UK	5	Women with fetal anomaly	10
USA	2	Women and their partner	5
Brazil	1	Health professional	1
Portugal	1	Terminating or continuing the pregnancy (Totals)	16
Iran	1	Terminating	7
Sweden	1	Continuing	3
Egypt	1	Both	6
Ireland	1	Place for data collection (Totals)	16
China	1	Care setting	6
Sri Lanka	1	Online	8
Germany	1	Convenient place for participants	1
Study design (Totals)	16	Not reported	1
Qualitative study	8	Coping instruments (Totals)	8
Quantitative study	7	Brief COPE	3
Mixed method	1	CBPS^*^	1
Sample size (Totals)	16	COPE^*^	1
< 50	12	G-QCP^*^	1
50–100	1	Kidcope scale	1
>100	3	Intrinsic religiosity scale	1

^*^CBPS, Coping Based on Problems Scale.

^*^COPE, Coping Operations Preference Enquiry.

^*^G-QCP, German Version of a Questionnaire on Coping Processes.

### Quality appraisal: Quantitative studies

The quality assessment details of cross-sectional studies are presented in Figure 2. A total of five studies did not identify the confounding factors and state strategies to deal with confounding factors, which may cause confounding bias. A few studies did not present the validity and reliability of measurements clearly (participant, exposure, or outcome), potentially leading to measurement bias. Statistical methods were unclear in two studies, probably causing bias during analysis.

### Quality appraisal: Qualitative studies

The quality assessment details of qualitative studies are presented in Figure 3. The main bias came for researchers because seven studies did not clarify the researcher's cultural and theoretical influence on the study, and six studies did not address the potential influence and relationship between the researcher and the study participants. The congruity between the research methodology and the representation and analysis of data was unclear in one study, and the representation of participants and their voices was unclear in two studies, which probably caused performance bias. One study did not report ethics approval.

### Coping strategies in quantitative studies

#### The measurement tools of the coping strategies

The details of coping strategies included in quantitative studies (*n* = 7) and the quantitative part of the mixed-method study (*n* = 1) are presented in Table 4. A total of five questionnaires (Brief COPE, CBPS, COPE, G-QCP, and the Kidcope Scale) were used to measure the coping strategies of women after being diagnosed with fetal anomaly pregnancy, and one questionnaire just measured intrinsic religiosity. There are 52 coping strategies in total after removing the duplicates. Acceptance, emotional support, active coping, planning, resignation, avoidant, problem-solving, positive reframe, self-distraction, positive reinterpretation, and growth were reported to be highly frequent coping strategies. However, two studies mainly focused on religiosity. The used dimension names include problem-focused coping, emotion-focused coping, religiosity/wishful thinking-focused coping, social support-focused coping, adaptive coping strategies, maladaptive coping strategies, active coping strategies, avoidant coping strategies, negative coping strategies, positive emotional attitude/distance, and negative emotional attitude/disapproval. Three studies used Brief COPE as their measurement tool, but different items were removed in each research. The other five questionnaires were separately used in five studies. Therefore, it is difficult to compare results with each other.

**Table 4:** Description of coping strategies included quantitative studies (*n* = 7) and the quantitative part of mixed-method study (*n* = 1).

**Measuring tool**	**Reference**	**Number of items**	**Coping strategies**	**Dimension of the tool**	**Data analysis about coping strategies**
CBPS^*^	Martins et al. (39)	45	No	Problem-focused coping; Emotion-focused coping; Religiosity/ wishful thinking-focused coping; Social support-focused coping	There was a positive correlation between practical religiosity scores and coping score.
Brief COPE	Lafarge et al. (4)	26	Self-distraction; active coping; denial; substance use; emotional support; instrumental support; behavioral disengagement; venting; positive reframing; planning; acceptance; use of religion; self-blame; (humor was deleted)	Adaptive coping strategies (positive reframing, acceptance); Maladaptive coping strategies (denial, self-blame); Other coping strategies.	Acceptance, emotional support, active coping and planning were the mainly used coping strategies. Adaptive coping strategies were negatively correlated with grief. Maladaptive coping strategies were positively correlated with grief.
Kidcope Scale	Shata et al. (42)	10	Problem solving; cognitive restructuring; emotional regulation; social support; distraction; social withdrawal; wishful thinking; resignation; self-criticism; blaming the husband	Active coping strategies; Avoidant coping strategies; Negative coping strategies	Participants used resignation, avoidant and problem solving high frequently, resignation and social support were high effectively, active coping strategies were significantly more effective than avoidant coping strategies.
Brief COPE	Nazare et al. (44)	20	Active coping; denial; emotional support; instrumental support; venting; positive reframing; acceptance; use of religion; self-blame; humor (planning, self-distraction, substance use, behavioral disengagement was deleted)	No	Acceptance, positive reframe, active coping and emotional support were the top four coping strategies. For men, grief was positively predicted by instrumental support and their partner's active coping. For women, grief was positively predicted by denial, self-blame and their partner's self-blame.
Brief COPE	Lafarge et al. (6)	26	Self-distraction; active coping; substance use; emotional support; instrumental support; behavioral disengagement; venting; positive reframing; planning; acceptance; use of religion; self-blame; (denial and humor were deleted)	Adaptive coping strategies (positive reframing, acceptance); Maladaptive coping strategies (Behavioral disengagement, self-blame)	Acceptance, emotional support, active coping, planning and self-distraction were the mainly used coping strategies. Grief was positively predicted by self-blame, behavioral disengagement, venting, planning and religion, negatively predicted by acceptance and positive reframing.
COPE^*^	Horsch et al. (7)	14	Active coping; planning; suppression of competing activities; restraint coping; seeking support for instrumental reasons; seeking social support for emotional reasons; positive reinterpretation and growth; acceptance; turning to religion; denial 3	Problem-focused coping; Emotion-focused coping	Acceptance, seeking social support for emotional reasons, positive reinterpretation and growth, and active coping were the top four coping strategies. Emotion-focused coping negatively correlated with the score of Hospital Anxiety and Depression Scale. Problem-focused coping did not associate with the score.
Intrinsic Religiosity Scale	Cowchock et al. (45)	Unclear	Intrinsic religiosity	No	Intrinsic religiosity negatively correlated with grief.
G-QCP^*^	Brisch et al. (8)	Unclear	Valorisation; humor; dissimulation; optimism; distraction; acceptance and/or stoicism; passive cooperation; self-blame; maintain composure; rumination; retreat; emotional relief; quarreling with one's fate; aggravation; resignation and/or fatalism; altruism; blaming others; isolation and/or suppression of feelings; distracting activity; analysis of problem; active avoidance; linking with others in similar situation; religiosity; emotional support; attribution of sense; compensation; active intervention; constructive activity; maintaining a sense of proportion; concentration and/or relaxation	Positive emotional attitude/ distance; Negative emotional attitude/ disapproval; Active coping	Positive emotional attitude negatively correlated with anxiety. Negative emotional attitude positively correlated with anxiety. Active coping had no significant correlation with anxiety.rh8

^*^CBPS, Coping Based on Problems Scale.

^*^COPE, Coping Operations Preference Enquiry.

^*^G-QCP, German Version of a Questionnaire on Coping Processes.

#### The relationship between coping strategies and mental stress is explored

Notably, five out of eight quantitative studies explored the coping mechanisms of women with fetal anomalies by analyzing their relationships with mental distress, such as grief, depression, or anxiety (4–8). Three studies analyzed the relationship between the dimensions of coping and mental distress; the other two analyzed each strategy and mental distress. The variables, outcomes, or results of these studies are inconsistent with each other (see details in Table 4). In one study, acceptance was grouped into adaptive coping strategies and denial was grouped into maladaptive coping strategies, and these two dimensions had opposite effects on grief (4). Otherwise, in another study, both acceptance and denial were grouped into emotion-focused coping, which negatively correlated with the score on the Hospital Anxiety and Depression Scale (7). Although the same questionnaire was used in two studies and explored each coping strategy's effect on grief, the results were still inconsistent in addition to self-blame (5, 6).

### Coping strategies in qualitative studies

**Figure 4:**
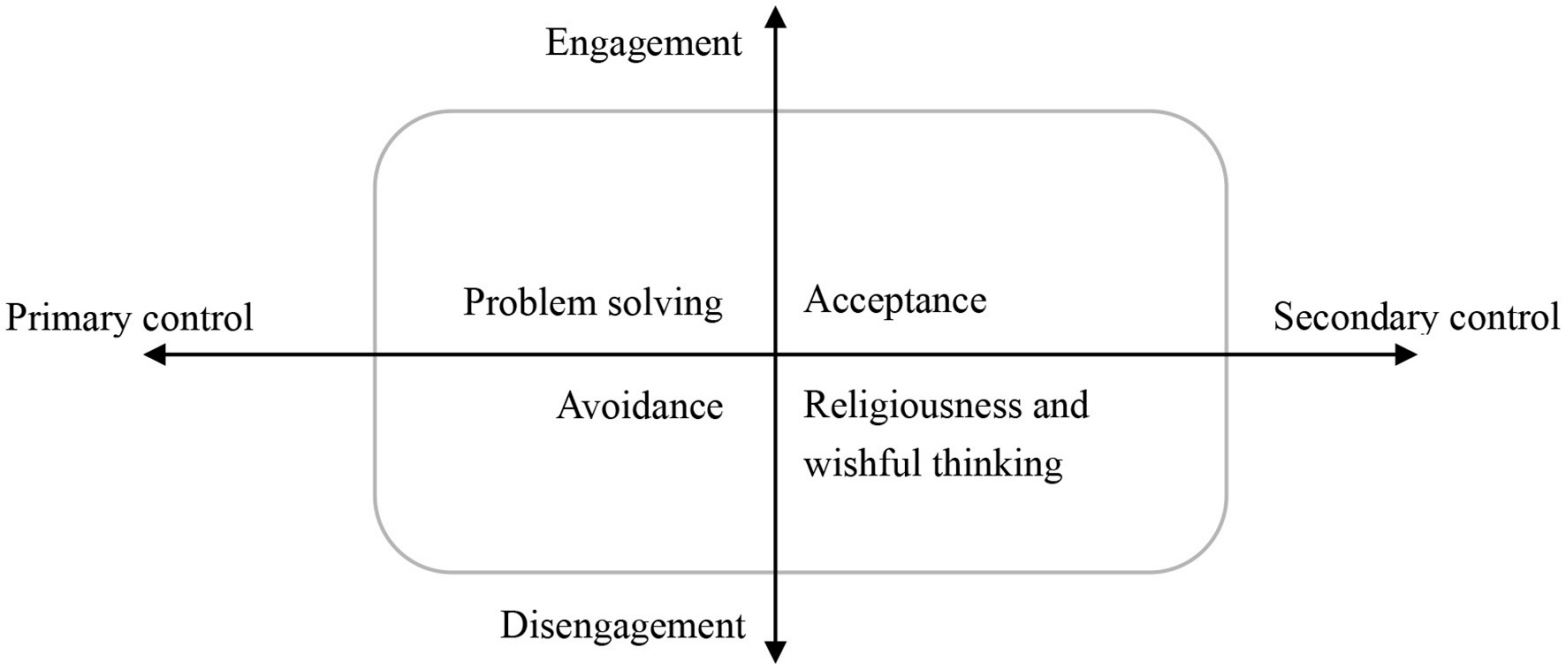
Categories of coping strategies for women with a fetal anomaly.

In these eight qualitative studies, women's coping strategies were the main topics of four studies (40, 46, 52, 53) and part topics of the other four studies (41, 43, 47, 48). The main topic of the mixed-method study was coping strategies, but qualitative research was part of it (42). We used a hierarchical model with two distinction elements (engagement vs. disengagement; primary control vs. secondary control) to present the coping strategies of women with a diagnosis of a fetal anomaly during pregnancy (Figure 4) (27, 28). Each of these elements formed an axis, and the two axes intersected (30); then, an intersection of these two elements resulted in four coping strategies (Figure 4): problem-solving (engagement and primary control coping), acceptance (engagement and secondary control coping), avoidance (disengagement and primary control coping), and religiousness and wishful thinking (disengagement and secondary control coping).

#### Engagement and primary control coping: problem-solving

This strategy was regarded as a coping strategy for women to have control over the situation (41, 52). Women tried to focus on the medical procedure for problem-solving, including asking for help from medical professionals about their baby's medical condition and development, being involved in healthcare decisions, and expressing individual requirements (41, 42, 52, 53). Seeking information and support was used frequently to control the situation. After the shock of diagnosis, many women expressed a significant need for detailed information (41). They needed the information to cope with the uncertainty, be actively involved in medical decisions, and continue the management of fetal wellbeing (46). They tried to find more information by searching on the internet, reading books, visiting different doctors, performing various diagnostic tests and sonography, and seeking peer experiences (40). Some women sought social support to decrease the high level of stress (40), including emotional support (40) and practical support (such as help with babysitting and chores) (41). Women sought not only individual-based support from partners, other family members, friends, and other women with the same experience (40) but also professional-based support (52, 53).

#### Engagement and secondary control coping: Acceptance

Acceptance was considered a coping strategy, including actively accepting the situation and acknowledging the baby (52, 53). In McKechnie et al. (43) study, some expectant parents chose to claim their child as their own. They said “*It's your kid. You're the parent of that kid that has the birth defect. And it's just such a hard thing to just do……*” Some others chose to continue the routine as if this was a normal pregnancy. They came to realize what they could or could not know about the diagnosis and accepted a conclusion about the diagnosis (43). In acceptance and commitment therapy, acceptance “involves the active and aware embrace of those private events occasioned by one's diagnosis without unnecessary attempts to change their frequency or form, especially when doing so would cause psychological harm” (54). Some women tried to embrace the diagnosis of fetal anomaly by freely expressing their feelings (41, 42). They expressed their thoughts and feeling to peers, families, friends, and health professionals. They said “*With all that I have, I can't keep calm to avoid getting depressed. I try to talk with all people around me”* (42).

#### Disengagement and primary control coping: avoidance

Avoiding the situation was described as a coping strategy of women with a diagnosis of a fetal anomaly during pregnancy nearly in all qualitative studies (40, 41, 43, 46, 48, 52, 53, 55) and supported by a mixed-method study (42). Some women were reluctant to receive negative information (40, 41, 46, 52). Some women distanced themselves from pregnancy, fetuses, other pregnant women, and young babies (43, 48, 53). Some recalled drinking heavily (53). Others focused their attention elsewhere (41), such as another pregnancy (52), healthy children (47), getting back to a routine, and trying to function as normally as possible (53). They avoided social situations because people treated pregnant women like public property, assuming a healthy baby (40, 55).

#### Disengagement and secondary control coping: religiousness and wishful thinking

Religiousness and wishful thinking coping mean believing in God, divine providence, and reliance on God's power. It contained three subcategories: praying, acceptance of destiny, and reliance on faith (40). Women hoped for a miracle to save their expected child (41). They had wishful thinking and said, “*we chose to continue the pregnancy, and we hoped that it wouldn't deteriorate*.” (42) They engaged in religious rites and believed in miraculous powers, hoping religious rites would beat the anomaly or prove the diagnosis wrong (47, 48, 52). The results showed that the belief in God had roots in the mother's mind so they understood it as divine providence and an opportunity to be examined in difficulties. Some women resigned to divine providence (48), “I told my husband that God handled everything….” They believed that if God gave them a problem, he also gave them the ability to tolerate it (40). The resignation was described as accepting their situation and making peace with their fate (42).

## Discussion

This scoping review presented the results of 16 studies to describe the coping strategies of women with a diagnosis of a fetal anomaly during pregnancy. A total of 52 coping strategies were collected in the quantitative studies using five questionnaires. The results of the main coping strategies of women or the correlation between coping strategy and mental distress were inconsistent and incomparable. All the coping strategies in qualitative studies were synthesized based on a meta-ethnographic approach. After constantly comparing with previous literature and theoretical frameworks, an intersection of two elements (engagement vs. disengagement and primary control vs. secondary control) was presented. This intersection classified the coping strategies into four categories: problem-solving, acceptance, avoidance, religiousness, and wishful thinking.

To our knowledge, this is the first review of the coping strategies of women with a diagnosis of a fetal anomaly during pregnancy. We identified five coping questionnaires used in seven quantitative studies in this review. Brief COPE was used in two different studies, but the items were different. Therefore, the results of coping strategies of women with a diagnosis of a fetal anomaly during pregnancy were inconsistent and incomparable. There were different measurements, dimensions, or items even when using the same questionnaire. Some studies failed to report the validity and reliability of the questionnaire (8, 39, 42). Some others categorized coping strategies into dimensions without factors analysis reporting (4, 7). No gold standard for measuring coping strategies of women with a diagnosis of a fetal anomaly during pregnancy has been established. Similar results were reported in reviews of other populations' coping, like caregivers of children with special illnesses (25, 26). The study of coping strategies attracted the wide attention of researchers because the ability to cope with stressful events played a primary role in reducing the risk for mental distress (56). More than 400 coping strategies were identified in one review across childhood, adolescence, and adulthood (24); it also reported that little consistency appeared across different measures and studies (28). Furthermore, all five questionnaires in this review were used to investigate coping strategies under general stress, and not used for women with a diagnosis of a fetal anomaly during pregnancy. Coping strategies in this special situation should be different from other situations (33). Therefore, we considered an appropriate measurement should be necessary for the research of coping in women diagnosed with fetal anomaly pregnancy.

After analyzing the results of qualitative studies, we classified the coping strategies with two distinct elements. In previous studies, authors also tried to categorize coping strategies into problem-focused and emotion-focused (39), adaptive and maladaptive (6), and positive and negative (8), but each of them cannot cover all the coping strategies that were being used. One study reported three coping categories: active, avoidant, and negative (42). However, it is confusing how avoidant and social withdrawal causes more negative outcomes in these women (57). Another study used problem-focused and emotion-focused to categorize all coping strategies (7). However, both acceptance and denial, whose effects were opposite in other studies, were in the same dimension. The results were consistent with the previous review, which recommended hierarchical systems for coping categories because all coping methods were multidimensional (24). Several hierarchical models of coping were reported in other populations (29–31). In this review, after synthesizing and constantly comparing, we used the intersection of engagement vs. disengagement and primary control vs. secondary control to distinguish coping's direction (toward or away from stressors) and action (influences objective events or adapt oneself to the events) features for women with fetal anomaly during pregnancy diagnosis. It should be helpful for researchers to understand their coping strategies more clearly.

## Limitations

There are several limitations in this review. First, to comprehensively explore the current research on the coping strategies of women after being diagnosed with fetal anomaly pregnancy, we did not exclude the studies without reporting reliability and validity of the coping questionnaire, confidence intervals, and odds ratio of regression analysis. Thus, caution should be used when interpreting the results of these surveys and this review. We included all studies that recruited women continuing or terminating their fetal anomaly pregnancy and their partners or healthcare providers. Therefore, the results of this review should be affirmed in future research for pregnant women, their partners, and healthcare providers. In addition, different groups of study participants might skew the accuracy of this review. Finally, only English and Chinese articles were included; however, we did not limit the language during article searching. Therefore, the search terminology or terms should be revised to recover relative studies in future.

### Implications of the findings for clinical practice

The findings of this review identified several implications for clinical practices. All the participants, including women, family caregivers, and healthcare providers, can understand the coping strategies used by women with fetal anomalies from this review. Although coping strategies and the effects are waiting to be explored and verified, this should provide information for healthcare providers to offer appropriate support (58). A better understanding of the coping strategies would be a foundation for improving the health of women with a fetal anomaly.

### Implications of the findings for future research

This review pointed out several significant studies on coping strategies of women with a diagnosis of a fetal anomaly during pregnancy: (1) establishing a special measurement (questionnaire or scale) to evaluate the coping strategies and analyze the correlation with mental stress; (2) using exploring or confirming factors analysis to demonstrate the coping strategies and their factors; (3) investigating the influence factors of coping strategies; and (4) exploring effective interventions for coping strategies that would decrease the incidence of mental stress.

## Conclusion

This scoping review identified the coping strategies of women with a diagnosis of a fetal anomaly during pregnancy from quantitative, qualitative, and mixed-method studies. Coping strategies included acceptance, emotional support, active coping, planning, resignation, avoidance, problem-solving, positive reframe, self-distraction, positive reinterpretation, and growth, which were reported to be used highly frequently. However, the results of quantitative studies were inconsistent and incomparable. Based on the synthesis of the qualitative results, the coping strategies have been classified into four categories shown in an intersection: problem-solving (engagement and primary control coping), acceptance (engagement and secondary control coping), avoidance (disengagement and primary control coping), and religiousness and wishful thinking (disengagement and secondary control coping). Establishing a valid and reliable measurement would be necessary for future studies on the coping of women with a diagnosis of fetal anomaly pregnancy. The result of this synthesis provides a clear framework for future research.

## Author contributions

HH and CQ: study design. TZ and YL: data collection. TZ, HP, and JX: data analysis. W-TC and QH: study supervision. TZ and W-TC: manuscript writing. HH, CQ, and TZ: critical revisions for important intellectual content. All authors contributed to the article and approved the submitted version.
