# Supplementary material for: Coping strategies following the diagnosis of a fetal anomaly: A scoping review
Source: Front Public Health. 2023 Apr 6;11:1055562. doi: 10.3389/fpubh.2023.1055562 (PMC10118031; doi:10.3389/fpubh.2023.1055562)
Supplement: Supplementary file 1 [file Table_1.DOCX]

| **Table# Search Strategies** | | |
| --- | --- | --- |
| Database | Search Formula | Results |
| 1. Web of Science | #1 ((((((TS=(“Fetal Malformation*”)) OR TS=("Fetal Anomal*")) OR TS=("Congenital Abnormalit*")) OR TS=("Congenital Defect*")) OR TS=("Congenital Deformit*")) OR TS=("Prenatal Diagnosis")) OR TS=("Fetal Death") | 169,109 |
|  | #2 ((((((((TS=("Psychological Adaptation")) OR TS=("Psychological Adjustment")) OR TS=("Coping Behavior*")) OR TS=("Coping Skill*")) OR TS=("Coping Strateg*")) OR TS=("Emotion* Regulation*")) OR TS=("Adaptive Behavior*")) OR TS=("Emotion* Adjustment")) OR TS=("Emotion* Adaptation") | 96,112 |
|  | #1 AND #2 | 139 |
| 2. CINAHL | S1 TI ( “Fetal Malformation*” OR "Fetal Anomal*" OR "Congenital Abnormalit*" OR "Congenital Defect*" OR "Congenital Deformit*" OR "Prenatal Diagnosis" OR "Fetal Death" ) OR AB ( “Fetal Malformation*” OR "Fetal Anomal*" OR "Congenital Abnormalit*" OR "Congenital Defect*" OR "Congenital Deformit*" OR "Prenatal Diagnosis" OR "Fetal Death" ) OR MH ( "Hereditary Diseases" OR "Fetal Abnormalities" ) | 14,628 |
|  | S2 TI ( "Psychological Adaptation" OR "Psychological Adjustment" OR "Coping Behavior*" OR "Coping Skill*" OR "Coping Strateg*" OR "Emotion* Regulation*" OR "Adaptive Behavior*" OR "Emotion* Adjustment" OR "Emotion* Adaptation" ) OR AB ( "Psychological Adaptation" OR "Psychological Adjustment" OR "Coping Behavior*" OR "Coping Skill*" OR "Coping Strateg*" OR "Emotion* Regulation*" OR "Adaptive Behavior*" OR "Emotion* Adjustment" OR "Emotion* Adaptation" ) OR MH ( coping OR "Adaptation, Psychological" OR "Emotional Regulations" ) | 78,816 |
|  | S3 S1 AND S2 | 142 |
| 3. EBSCO PsycARTICLES | S1 TI ( “Fetal Malformation*” OR "Fetal Anomal*" OR "Congenital Abnormalit*" OR "Congenital Defect*" OR "Congenital Deformit*" OR "Prenatal Diagnosis" OR "Fetal Death" ) OR AB ( “Fetal Malformation*” OR "Fetal Anomal*" OR "Congenital Abnormalit*" OR "Congenital Defect*" OR "Congenital Deformit*" OR "Prenatal Diagnosis" OR "Fetal Death" ) | 577 |
|  | S2 TI ( "Psychological Adaptation" OR "Psychological Adjustment" OR "Coping Behavior*" OR "Coping Skill*" OR "Coping Strateg*" OR "Emotion* Regulation*" OR "Adaptive Behavior*" OR "Emotion* Adjustment" OR "Emotion* Adaptation" ) OR AB ( "Psychological Adaptation" OR "Psychological Adjustment" OR "Coping Behavior*" OR "Coping Skill*" OR "Coping Strateg*" OR "Emotion* Regulation*" OR "Adaptive Behavior*" OR "Emotion* Adjustment" OR "Emotion* Adaptation" ) | 10,718 |
|  | S3 S1 AND S2 | 6 |
| 4.Google Scholar | All fields:("fetal malformation" OR "fetal anomaly" OR "congenital abnormality" OR "congenital defect" OR "congenital deformity") AND ("Psychological Adaptation" OR "Psychological Adjustment" OR "coping behavior" OR "coping skill" OR "coping strategy" OR "adaptive behavior") | 539 |
| 5.Cochrane | #1 (“Fetal Malformation*” OR "Fetal Anomal*" OR "Congenital Abnormalit*" OR "Congenital Defect*" OR "Congenital Deformit*" OR "Prenatal Diagnosis" OR "Fetal Death"):ti,ab,kw (Word variations have been searched) | 1,305 |
|  | #2 MeSH descriptor: [Congenital Abnormalities] explode all trees | 7,935 |
|  | #3 MeSH descriptor: [Fetal Death] 1 tree(s) exploded | 451 |
|  | #4 MeSH descriptor: [Prenatal Diagnosis] explode all trees | 1,046 |
|  | #5 #1 OR #2 OR #3 OR #4 | 9,902 |
|  | #6 ("Psychological Adaptation" OR "Psychological Adjustment" OR "Coping Behavior*" OR "Coping Skill*" OR "Coping Strateg*" OR "Emotion* Regulation*" OR "Adaptive Behavior*" OR "Emotion* Adjustment" OR "Emotion* Adaptation"):ti,ab,kw (Word variations have been searched) | 8,087 |
|  | #7 MeSH descriptor: [Adaptation, Psychological] explode all trees | 6,261 |
|  | #8 MeSH descriptor: [Emotional Adjustment] explode all trees | 116 |
|  | #9 #6 OR #7 OR #8 | 12,994 |
|  | #10 #5 AND #9 | 69 |
| 6.PubMed | #1 "fetal malformation*"[Title/Abstract] OR "fetal anomal*"[Title/Abstract] OR "congenital abnormalit*"[Title/Abstract] OR "congenital defect*"[Title/Abstract] OR "congenital deformit*"[Title/Abstract] OR "Prenatal Diagnosis"[Title/Abstract] OR "Fetal Death"[Title/Abstract] | 52,394 |
|  | #2 "Psychological Adaptation"[Title/Abstract] OR "Psychological Adjustment"[Title/Abstract] OR "coping behavior*"[Title/Abstract] OR "coping skill*"[Title/Abstract] OR "coping strateg*"[Title/Abstract] OR "emotion regulation*"[Title/Abstract] OR "adaptive behavior*"[Title/Abstract] OR "emotion adjustment"[Title/Abstract] OR "emotion adaptation"[Title/Abstract] OR "adaptation, psychological"[MeSH Terms] OR "Emotional Regulation"[MeSH Terms] | 164,567 |
|  | #3 ("fetal malformation*"[Title/Abstract] OR "fetal anomal*"[Title/Abstract] OR "congenital abnormalit*"[Title/Abstract] OR "congenital defect*"[Title/Abstract] OR "congenital deformit*"[Title/Abstract] OR "Prenatal Diagnosis"[Title/Abstract] OR "Fetal Death"[Title/Abstract]) AND ("Psychological Adaptation"[Title/Abstract] OR "Psychological Adjustment"[Title/Abstract] OR "coping behavior*"[Title/Abstract] OR "coping skill*"[Title/Abstract] OR "coping strateg*"[Title/Abstract] OR "emotion regulation*"[Title/Abstract] OR "adaptive behavior*"[Title/Abstract] OR "emotion adjustment"[Title/Abstract] OR "emotion adaptation"[Title/Abstract] OR ("adaptation, psychological"[MeSH Terms] OR "Emotional Regulation"[MeSH Terms])) AND "journal article"[Publication Type] | 168 |
